# Moderate Genetic Diversity and Demographic Reduction in the Threatened Giant Anteater, *Myrmecophaga tridactyla*

**DOI:** 10.3389/fgene.2021.669350

**Published:** 2021-07-01

**Authors:** Carmen Elena Barragán-Ruiz, Rosane Silva-Santos, Bruno H. Saranholi, Arnaud L. J. Desbiez, Pedro Manoel Galetti

**Affiliations:** ^1^Departamento de Genética e Evolução, Universidade Federal de São Carlos, São Carlos, Brazil; ^2^Department of Life Sciences, Imperial College London, Ascot, United Kingdom; ^3^Instituto de Conservação de Animais Silvestres, Campo Grande, Brazil; ^4^Royal Zoological Society of Scotland, Edinburgh, United Kingdom; ^5^Instituto de Pesquisas Ecológicas, Nazaré Paulista, Brazil

**Keywords:** bottleneck, inbreeding, population size reduction, microsatellite markers (SSR), Xenarthra

## Abstract

In general, large mammal species with highly specialized feeding behavior and solitary habits are expected to suffer genetic consequences from habitat loss and fragmentation. To test this hypothesis, we analyzed the genetic diversity distribution of the threatened giant anteater inhabiting a human-modified landscape. We used 10 microsatellite loci to assess the genetic diversity and population structure of 107 giant anteaters sampled in the Brazilian Central-Western region. No genetic population structuring was observed in this region suggesting no gene flow restriction within the studied area. On the other hand, the moderate level of genetic diversity (Ho = 0.54), recent bottleneck detected and inbreeding (F_is_, 0.13; *p* ≤ 0.001) signatures suggest potential impacts on the genetic variation of this Xenarthra. Additionally, a previous demographic reduction was suggested. Thus, considering the increased human-promoted impacts across the entire area of distribution of the giant anteater, our results can illustrate the potential effects of these disturbances on the genetic variation, allowing us to request the long-term conservation of this emblematic species.

## Introduction

During the last decades, anthropogenic impacts have promoted habitat loss and fragmentation by extensive agriculture, urbanization, and highways and thus threaten biodiversity worldwide (Storfer et al., 2010; Haddad et al., 2015) including populations of wild animals. More and more, isolated populations are affected by decreasing population size (Reed and Frankham, 2003) and reduced gene flow (Haag et al., 2010; Oliveira and Hannibal, 2017) and become more sensitive to genetic drift effects (Reed and Frankham, 2003). Consequently, local genetic variation can be reduced, and genetic differentiation among populations increases, negatively impacting the long-term persistence of wild populations (Reed and Frankham, 2003). In this scenario, large mammals are the most threatened vertebrates affected by habitat loss and fragmentation, resulting in genetic variation loss (Lino et al., 2019).

Extant in several major biomes across Central and South America, the giant anteater, *Myrmecophaga tridactyla*, is a charismatic and large Xenarthra that has been suffering from human activities in several regions of its distribution area. Currently categorized as “Vulnerable” and with decreasing populations in the International Union for Conservation of Nature (IUCN) Red List (Miranda et al., 2015) and in the Brazilian Threatened Species List (Miranda et al., 2018), the giant anteater has disappeared in several areas of its original range (Bertassoni et al., 2014), mainly due to habitat reduction and fragmentation caused by anthropic activities (Bertassoni et al., 2014; Miranda et al., 2015). Its solitary habits, low fecundity, long gestation time, and relatively high generation time (Eisenberg and Redford, 1999) added to a specialist diet (McNab, 1984), making this species more vulnerable and threatened in anthropic scenarios (Desbiez et al., 2020). Within the distribution area of the giant anteater, mitochondrial haplogroups have been described, separating a population in the Amazon Forest from another group represented by individuals from the Cerrado and Pantanal biomes (Clozato et al., 2017). Although two studies using local populations have already been published, little is known about the consequences on the genetic variation in highly anthropized regions of these vulnerable animals in Brazil. A previous genetic study on anteaters in Central-Western Brazil evidenced a low genetic diversity and high inbreeding in a small local population inhabiting a protected area submitted to recurrent fire events (Collevatti et al., 2007). Conversely, another study in Central-Southern Brazil suggests high levels of genetic diversity in a regional geographic scale accompanied by spatial population differentiation (Sartori et al., 2020). Of note, all these previous studies focused on small local populations inhabiting protected areas or surrounding protected areas, and there is no genetic populational analysis evaluating this genetic information in a large-scale anthropized area.

The Brazilian Central-Western region, located on the southern edge of the distribution area of the giant anteater, has been undergoing an intense urbanization process with remarkable agriculture development (Brazilian Institute of Geography and Statistics (IBGE), 2020) and an increase in roads and highway constructions (Grilo et al., 2019). These landscape modifications are relatively recent and have mostly occurred during the last five decades (Brazilian Institute of Geography and Statistics (IBGE), 2020). In this context, we predicted that the giant anteaters living in this increasingly human-modified landscape would lose genetic diversity and show fragmented populations with reduced gene flow. Therefore, we tested the hypothesis that anteater populations inhabiting a large polygon in Central-Western Brazil will show reduced genetic diversity and signals of gene flow reduction among local populations. In addition, we hypothesized that a reduction in the effective population size of anteaters, due to the high loss of natural habitat, will be observed. This represents the first large population genetic study in giant anteaters.

## Materials and Methods

### Ethics Statements

The biological sampling authorization was obtained through the SISBIO-ICMBio (Authorization System and Biodiversity Information, Chico Mendes Institute for Biodiversity Conservation, Ministry of Environment, Brazil), under the number 53798-4. The research was approved by the Ethics Committee on the Animal Experimentation (CEUA/UFSCar) protocol number 1584280817, and the genetic resource access was registered under SisGen A9F8717.

### Study Area and Sampling

**FIGURE 1:**
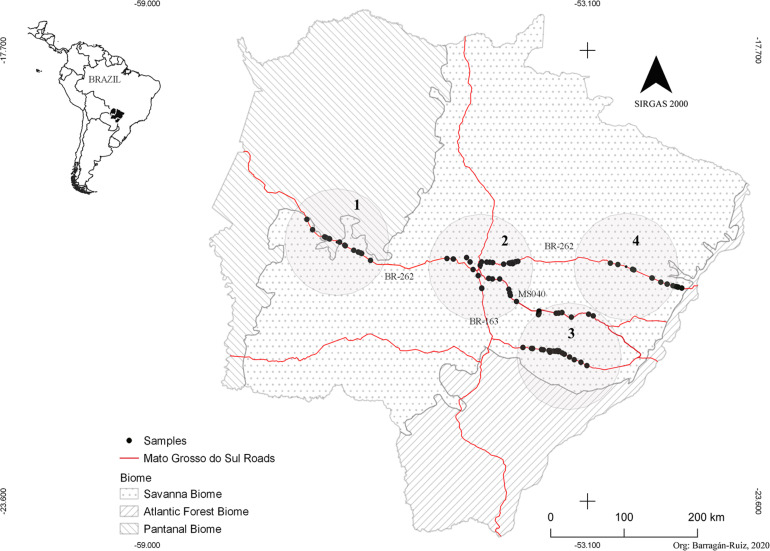
Geographic location of *Myrmecophaga tridactyla* individual sampled. Red lines represent the main Mato Grosso do Sul roads, and black dots are each individual.

The study was carried out in the Central-Western region of Brazil, comprising the biome Cerrado (Neotropical savanna) and transition areas with two other biomes, the Pantanal wetlands and inland Atlantic forest (Figure 1). In this area, agriculture has transformed the landscape into a mosaic of monocultures, mainly soy and sugarcane crops, and pasture with different degradation levels of natural vegetation (Brazilian Institute of Geography and Statistics (IBGE), 2020), besides urbanization, roads, and highways.

We collected a total of 107 tissue samples, comprising 66 samples from roadkill animals in four main roads crossing our study area and 41 samples obtained from captured wild animals (Figure 1). All tissue samples were conserved in 95% ethyl alcohol and stored in a freezer at −20°C. All samples were collected by the research project “Anteaters and Highways”^1^. The sample collection was conducted from April 2013 to February 2017. This sampling represents the largest range for a giant anteater population genetically evaluated so far. A detailed information related to each specimen sample is available in Supplementary Table 1.

### DNA Extraction and Genotyping Genetic Analysis

Total genomic DNA was extracted using the conventional phenol-chloroform protocol (Sambrook et al., 1989). The DNA quality was checked by electrophoresis on 1% agarose gel stained with Gel Red^TM^ (Biotium, Hayward, CA, United States).

A total of 10 microsatellite loci (Supplementary Table 2) were used for genotyping all the individuals. Five microsatellites (04, 07, 11, 13, and 20) were described for *M. tridactyla* (Garcia et al., 2005), and five heterologous primers (A9, B2, E3, G3, and H5) were developed for *Tamandua tetradactyla* (Clozato et al., 2014). We used a universal M13 primer fluorescent-labeled and an M13 complementary tail to the 5′ position of each forward primer (Schuelke, 2000) for genotyping each locus. The PCR reaction was performed in a final volume of 10 μl containing 1 U GoTaq DNA polymerase (Promega), 1 × buffer, 1.5 mM MgCl_2_, 0.20 mM deoxyribonucleotide triphosphates (dNTPs), 0.8 mg/ml bovine serum albumin (BSA), 2 pmol forward, and 8 pmol of reverse primers, 8 pmol M13 primers, and ∼30 ng of the target DNA. PCRs were conducted in two steps. PCRs were run with an initial denaturing step of 1 min at 94°C, followed by 20 cycles of 1 min at 94°C, 45 s at locus-specific annealing temperature (Supplementary Table 2), and 1 min at 72°C. In a second step, eight cycles of the 30 s at 94°C, 45 s at 53°C, and 45 s at 72°C were added, and a final extension for 20 min at 72°C. PCR products were checked on 2% agarose gel. Fragments were genotyped using an ABI3730XL automatic sequencer (Applied Biosystems, United States). Allele sizes were scored using internal standard ROX 550 and manually determined using Geneious R7 (Biomatters Ltd, New Zealand) (Kearse et al., 2012). The samples consistently producing not concordant or negative genotypes at a locus after three repetitions using different DNA aliquots were treated as missing data.

### Genetic Population Structuring and Genetic Diversity

The presence of null alleles and scoring errors due to allelic dropout and stutter peaks were checked using MICROCHECKER v. 2.2.3 (Van Oosterhout et al., 2004) and Oosterhout estimator. Genetic population structuring was investigated using different methods, in which our sampling was first organized in four 200-km diameter sampling areas, representing what we considered the main sampling areas, named hereafter sampling sites 1–4 (Figure 1). We used the Bayesian assignment analysis implemented in the STRUCTURE v. 2.3.3 software (Pritchard et al., 2000). The most likely number of clusters (K) was tested using the admixture model with sampling location as prior (LOCPRIOR) information, with 1,000,000 Markov chain Monte Carlo (MCMC) iterations, and each *K*-value (1–5) was tested with 10 replicates and burn-in at 1,000. We tested for K ranging from 1 to 5 because, for K determination based in the highest value of ΔK, following Evanno et al. (2005), it is necessary to use the maximum number expected for K (*K* = 4, in our case) plus 1. For ΔK estimation, we used the algorithm implemented in STRUCTURE HARVESTER (Earl and vonHoldt, 2012). We also verified the best K in STRUCTURE based on the Ln value according to Pritchard et al. (2000). We furthermore used the GENELAND package (Guillot et al., 2005), implemented in R Core Team (2017), to conduct a Bayesian spatial clustering model. GENELAND uses spatial location of the samples, which provides more support to clustering analyses, even when crypt patterns of population structuring occur (McManus et al., 2015) and can be especially helpful in the case of sparse sampling (Ball et al., 2010). In this analysis, we used the correlated frequency model, 1,000,000 MCMC iterations, and thinning and burn-in parameters set at 1,000 and 200, respectively. The tested group number was *K* = 1–4. The choice of K was based on the histogram of estimated K for each run, and the highest mean posterior density across replicates was considered the best K.

Population structuring was also evaluated by a multivariate approach using discriminant analysis of principal components (DAPC; Jombart et al., 2010) from the Adegenet package (Jombart, 2008), implemented in the R software (R Core Team, 2017), which do not make any assumption about the underlying population genetic model (Jombart, 2008).

To test the correlation between the genetic and geographic distances and check a possible sexual dispersion bias reported by Collevatti et al. (2007), we evaluated the presence of isolation by distance (IBD) using the Mantel test (Mantel, 1967). All individuals had the sex previously assigned by molecular identification using the protocol of Barragán-Ruiz et al. (2020) (sex individual information in Supplementary Table 1). The genetic similarity between pairs of individuals at several distance classes was assessed by a spatial autocorrelation analysis, using a 20-km distance class and a total of 50-km distance classes. The significance values were assessed using 9,999 permutations and 95% confidence intervals. A significant positive autocorrelation means that individuals at a given distance class are genetically more similar than randomly expected. Both Mantel test and the spatial autocorrelation analysis were carried out in the GenAlex v. 6.5.0 software (Smouse and Peakall, 2012).

The population was redefined according to the results concordantly obtained in all the genetic structuring analyses (*K* = 1), and the microsatellite loci were tested for linkage disequilibrium (LD) and Hardy–Weinberg equilibrium (HWE), using the exact test of Guol and Thompson (1992) for heterozygote deficit in GENEPOP v. 1.2 (Raymond and Rousset, 1994). For both LD and HWE tests, we estimated *p*-values using the Markov chain methods with 10,000 dememorization steps, 1,000 batches, and 10,000 iterations per batch. Sequential Bonferroni corrections were applied to correct for all multiple simultaneous comparisons (Rice, 1989).

Genetic diversity was estimated by the number of alleles (Na), effective number of alleles (Ae), observed (Ho), and expected heterozygosity (He) using GenAlex v. 6.5.0 (Peakall and Smouse, 2012). Allelic richness (AR, Leberg, 2002) and inbreeding coefficient (F_is_) (Weir and Cockerham, 1984) with the *p*-value for heterozygote excess (pL) and deficit (pS) were calculated for each locus using FSTAT v. 2.9.3.2 (Goudet, 1995). To verify a kinship effect in F_is_ values, we calculated different kinship estimators (*r*) (Queller and Goodnight, 1989; Ritland, 1996; Lynch and Ritland, 1999) among all individuals and within each sampling site. The *r*-values were calculated in the GenAlex v. 6.5.0 software (Peakall and Smouse, 2012). We calculated the polymorphic information content (PIC) using the Cervus 3.0.3 software (Slate et al., 2000).

### Genetic Diversity in Bottleneck Scenarios

To assess whether the current effective population size of giant anteater is sufficient to maintain the observed genetic variation over the next 100 years, we simulated future genetic diversity using the program BOTTLESIM v. 2.6 (Kuo and Janzen, 2003) that measured changes in genetic diversity assuming no selection, migration, and mutation. We verified changes in the genetic diversity parameters (observed number of alleles, effective number of alleles, observed and expected heterozygosity) under different population reduction scenarios, using as initial population size the effective population size obtained here. The future genetic diversity parameters were simulated over 100 years when retaining 100, 75, 50, 25, and 10% of the current effective population size. All simulation parameters were set as follows: degree of generation overlap = 100 (i.e., all individuals start with a random age value that is within the longevity limit), dioecy with random mating reproductive system, expected longevity = 15 years, age of reproductive maturation = 4 years, male/female ratio was set to 1:1 (parameters according to Desbiez et al., 2020), number of years simulated = 100 years, and number of iterations = 1,000.

### Demographic Changes

We measured the contemporary effective population size (Ne) using the linkage disequilibrium (LD) method (Waples and Do, 2010) and the jackknife resampling method to determine the effective population size with 95% confidence intervals. We calculated this parameter using the NeEstimator 2.0 software (Do et al., 2013).

To assess recent signatures of population size reduction, we used both the Wilcoxon test (Luikart and Cornuet, 1998) and *M*-ratio (Garza and Williamson, 2001). Wilcoxon test was done using the infinite alleles (IAM), stepwise mutation (SMM), and two-phase (TPM) mutation models in BOTTLENECK v. 1.2.02 (Cornuet and Luikart, 1996; Luikart and Cornuet, 1999). Wilcoxon test provides relatively high power to identity significative population size reduction signatures and can be applied to data sets with few polymorphic loci. For the TPM model, a variance of 30, probability of 90%, and 1,000 interactions were assumed. Genetic bottlenecks can also leave a signature in the ratio of the number of alleles and the allele size range (the *M*-ratio), where a bottleneck depletes the number of alleles faster than reducing allele size range of the microsatellite (Garza and Williamson, 2001). We calculated the *M-*ratio by *M* = k/r formula, where k is the number of alleles and r = S_max_ − S_min_ + 1 (S_max_ is the size of the largest allele, and S_min_ is the size of the smallest allele in the sample), using ARLEQUIN v 3.5 (Excoffier and Lischer, 2010). It was considered that *M* < 0.68 indicates a bottleneck, while *M* > 0.80 indicates no reduction in effective population size (Garza and Williamson, 2001).

### Scenario’s Test of Demographic History

We investigated historical changes in the effective population size using approximate Bayesian computation (ABC) implemented in DIYABC (Cornuet et al., 2010). We designed our ABC analysis in three steps: (1) a preliminary analysis to determine proper prior intervals, (2) an analysis to evaluate the suitability of each summary statistic, and (3) a final analysis to quantify the relative posterior probabilities of the models. We assessed the population size changes on the giant anteater population through the time, testing three different scenarios (Figure 3): (1) the population size has been stable during the time (null hypothesis, Na = Nr, where Na is the ancestral effective population size, and Nr is the recent effective population size); (2) the population experiencing a reduction in the population size at coalescent time t (bottleneck event, Nr < Na); and (3) there was an expansion that led to an increase in the effective population size of the giant anteater (Na > Nr). In ABC, competing population scenarios are simulated, and statistical tests are then used to assess which scenario better fits the observed data. We performed one million simulations per scenario. The prior settings for all parameters (effective population size, time, and mutation rate) are shown in Supplementary Table 3. In DIYABC analysis, the generation time of a given species is considered the elapsed time between the birth of an individual and the birth of its first offspring (Cornuet et al., 2014), which was assumed as 4 years in the giant anteater, according to Desbiez et al. (2020). The summary statistics employed were the mean number of alleles, mean expected and observed heterozygosity, and mean allele size variance. We analyzed each locus separately for increasing the total number of summary statistics and improving the simulation results (Cornuet et al., 2014). Thus, we had 30 summary statistics once each microsatellite was considered a distinct group to run the analysis. The reliability of scenarios was visualized through principal component analysis.

To obtain the best fit scenario, the posterior probability (PP) for each scenario was estimated by logistic regression on 1% of the simulated dataset closest to the empirical data. For the scenario with high PP, we evaluated the confidence in the scenario choice estimating the posterior predictive global error using 1,000 pseudo-observed dataset for the logistic regression approach. To assess the precision for each estimated parameter, we calculated the relative median of the absolute error (RMAE) (Cornuet et al., 2010). The best model was tested by comparing the summary statistics (mean allele size variance and mean Garza–Williamson’s M index) between the observed and simulated datasets.

## Results

### Population Genetic Structuring and Genetic Diversity

The PIC values for each locus were higher than 0.5, with a mean value of 0.53 (Table 1), indicating that our multiloci panel was highly informative and adequate for population genetic analyses in *M. tridactyla*.

**TABLE 1:** Summary information on the 10 microsatellite loci used in *Myrmecophaga tridactyla.*

Locus	N	Na	Ae	AR	Ho	He	*p*-values	F_is_	Null alleles	PIC	*M*-ratio
4*	107	4	2.06	3.99	0.27	0.51	0.0162	**0.47**	0.2213	0.42	0.44
7	74	9	6.03	9.00	0.84	0.83	0.4102	0.00	−0.0029	0.81	0.52
13	98	6	3.56	6.00	0.82	0.72	0.9815	**−0.13**	−0.0734	0.68	0.46
11*	96	8	3.02	7.49	0.64	0.67	0.0087	0.05	0.0145	0.63	0.22
20	97	8	5.56	7.76	0.80	0.82	0.4610	0.02	0.0114	0.80	0.47
A9*	82	5	3.25	4.99	0.56	0.69	0.0052	**0.19**	0.0844	0.64	0.55
B2	98	3	2.08	3.00	0.61	0.52	0.9745	−0.18	−0.1296	0.46	0.60
E3	99	4	1.61	3.75	0.37	0.38	0.1298	0.01	−0.0180	0.35	0.26
G38	83	3	1.80	3.00	0.46	0.44	**0.0001**	−0.03	−0.0275	0.37	0.08
H5*	79	5	2.04	5.00	0.05	0.51	**0.0000**	**0.90**	0.3715	0.47	0.45
Mean	91	5.5	3.10	5.40	0.54	0.61	–	**0.13**	–	0.56	–

Loci name, number of individuals (N), number of alleles per locus (Na), effective number of alleles (Ae), allelic richness (AR), observed and expected heterozygosity (Ho and He, respectively), global estimate of F_*is*_, results for the null allele test, polymorphic information content (PIC), and the Garza–Williamson index, the number of alleles ratio to range in allele size (M-ratio). Bold values indicate significative F_*is*_ values (≤0.001). *Loci with Hardy–Weinberg equilibrium (HWE) after Bonferroni correction (p ≤ 0.005).

**FIGURE 2:**
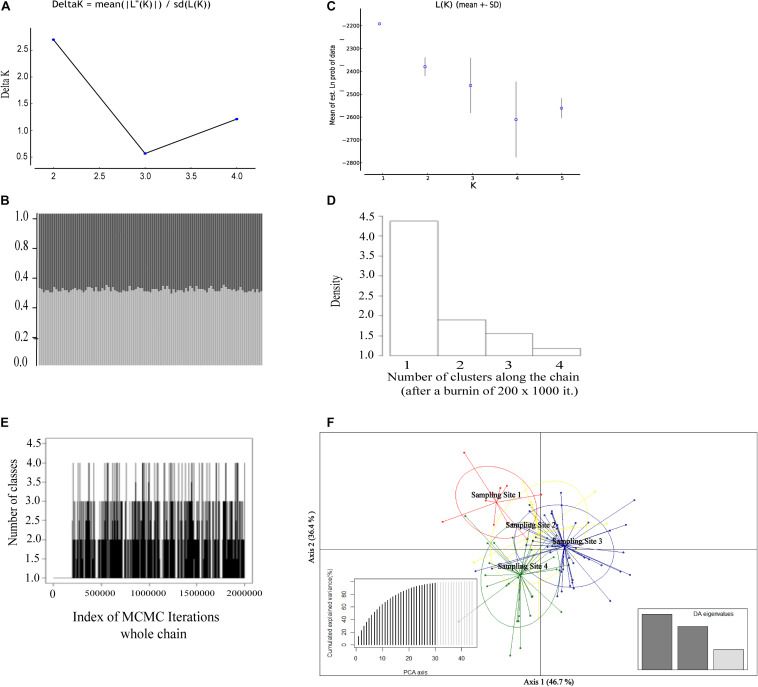
Genetic structure of 107 *Myrmecophaga tridactyla* specimens assessed by different approaches based on 10 microsatellite loci. **(A)** Population structure results (*K* = 2) based on the ΔK statistic (Evanno et al., 2005). **(B)** Graphical representation of *K* = 2 from structure results based on ΔK statistic (Evanno et al., 2005). Each vertical bar represents an individual and each color (light gray and dark gray) represents the posterior probability of the individuals belonging to that cluster. **(C)** Graphical representation of *K* = 1 from structure result based on the Ln value (Pritchard et al., 2000). **(D)** The number of clustering among the chain from GENELAND. **(E)** Plot of the number of populations simulated from the posterior distribution with GENELAND, indicating *K* = 1 as the most frequent result. **(F)** Results of the discriminant analysis of principal components (DAPC) showing the scatterplot of the first two principal components and DA% for each axis.

All clustering approaches were agreeing to define a single genetic population for the giant anteater individuals analyzed (Figure 2). Although *K* = 2 was obtained according to the Evanno et al. (2005) criterion [LnP (K) = −2,251.07 and ΔK = 2.69; Figure 2A), the graphic of individual assignment showed similar probability for a given individual to belong to one or another population (Figure 2B), supporting an absence of population structuring and indicating that the K definition based on ΔK is not able to define de minimum K (*K* = 1). In turn, the best K based on the Ln value revealed *K* = 1 (Figure 2C). The absence of population structuring was also inferred by the spatial analysis in GENELAND (*K* = 1; Figures 2D,E). Similarly, the multivariate analysis (DAPC) showed a clear overlap among the sampling sites tested, reinforcing the findings of a single population pattern (Figure 2F).

**FIGURE 3:**
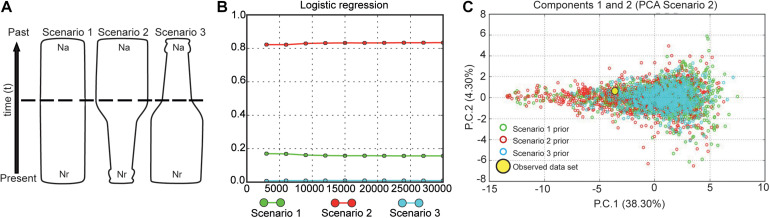
Possible demographic history scenarios for the *Myrmecophaga tridactyla* population. **(A)** Representation of three demographic scenarios evaluated by DIYABC. Legend: the areas of the figures represent changes in population size through time. Effective population size (Ne) is represented by Na (ancestral effective population size) and Nr (recent effective population size). The time, t, in number of generations. Scenario 1 without a change in an ancestral population experiencing (null hypothesis); scenario 2 with a change in Na at time t, representing a bottleneck event, Nr < Nr; and scenario 3 with a change in Ne at time t, representing an expansion event, Nr > Na. **(B)** Posterior probabilities of the three scenarios obtained by logistic regression of 1% of the closest simulated datasets. The most probable demographic scenario for *M. tridactyla* population was a historical bottleneck. Posterior probability of each scenario in the y- and x-axes indicates the number of simulated data closest to observed data. **(C)** Graphic of principal components analysis (PCA) generated in DIYABC displaying the fit between scenarios simulated and our dataset.

The analyses of genetic spatial autocorrelation showed no significant autocorrelation between individuals in all measured distances (*p* ≤ 0.05), even when females and males were separately analyzed (Supplementary Figure 1 and Figures 1A, 2A, 3A). The Mantel test showed no association of genetic variation and geographic distance, neither considering the total of individuals nor each gender separately (Supplementary Figure 1 and Figures 1B, 2B, 3B).

The subsequent genetic analyses considering all individuals belonging to a single large population revealed no significant linkage disequilibrium, although deviation from HWE (*p* ≤ 0.005 after Bonferroni correction) occurred in five loci, with locus 4, A9, and H5 showing heterozygote deficit. Locus H5 also showed high amount (37%) of null alleles (Table 1). We analyzed our dataset with and without this latter locus, and we founded similar results. Thus, all analyses included the complete set of 10 microsatellites.

A total of 55 alleles were obtained in the 107 samples. The number of alleles/locus ranged from three (B2 and G3) to eight (11 and 20) with a mean of 5.5, and the mean number of effective alleles (Ae) was 3.10 (Table 1). Mean observed heterozygosity (Ho) was 0.54 (ranging from 0.05 to 0.84), and the mean expected heterozygosity (He) was 0.61 (ranging from 0.38 to 0.83). The F_is_ values ranged from −0.28 to 0.47, with a statistically significant mean value of 0.13 (*p* ≤ 0.001). Low relatedness level was found among the individuals (see *r*-values in Supplementary Table 4).

### Genetic Diversity in Bottleneck Scenarios

**FIGURE 4:**
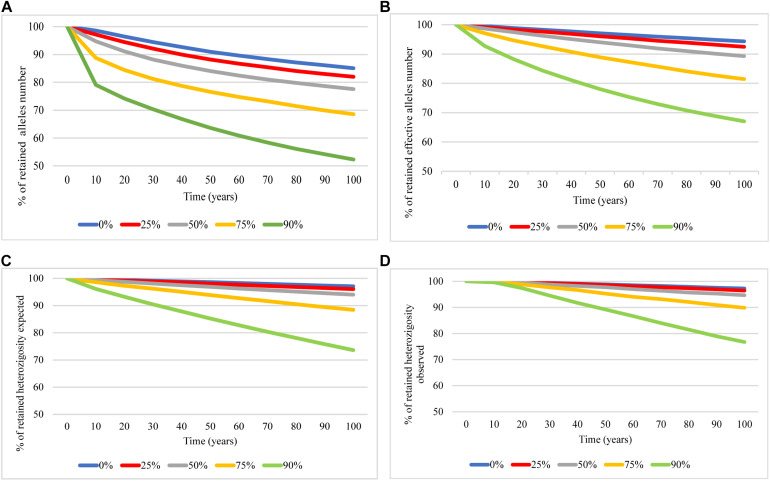
Predicted genetic diversity in *Myrmecophaga tridactyla* over next 100 years when retained 100% (blue), 75% (red), 50% (gray), 25% (yellow), and 10% (green) of the current effective population size using BOTTLESIM program. **(A)** Number of alleles. **(B)** Number of effective alleles. **(C)** Expected heterozygosity. **(D)** Observed heterozygosity.

The prediction of future genetic diversity based on BOTTLESIM simulations projected a genetic diversity decrease in the next 100 years in all tested scenarios. Overall, the genetic diversity reduction was directly affected by the bottleneck intensity tested. The observed allele number and effective allele number declined at a faster rate than expected and observed heterozygosity (Figure 4). The predicted future simulation showed a decline of about 15% in the number of alleles, 6% in the effective number of alleles, and 3% of expected and observed heterozygosity in the giant anteater population studied even at the retention of 100% of individuals during the next 100 years (blue line, Figure 4). The genetic diversity decline in the next 100 years will be sharper as the bottleneck intensity is higher.

### Effective Population Size Variation

The effective population size (Ne) estimate was 375.5 (CI = 80.2 − ∞; *p* < 0.05). Signs of population reduction were significant for the TPM model (*p* = 0.0048) in the bottleneck analysis, and the *M*-ratio also showed a signal of population reduction (*M* = 0.39).

The scenario that best explained our data was scenario 2, indicating that the giant anteater experienced a reduction in the effective population size in the past. This hypothetical scenario showed a posterior probability of 0.8339 with a posterior error rate of 0.304 (Supplementary Table 5). All our RMAE values were < 2 (Nr = 0.191, Na = 0.316, and *t* = 0.293), indicating that all parameters estimated were reliable, suggesting a high confidence for scenario 2 (Figure 3). At this scenario, the effective population size Nr and Na had average values of 1,119 (95% CI = 660–2,040) and 6,370 (95% CI = 2,130–9,830), respectively. When we applied the model checking (Supplementary Figure 2), we observed that our best scenario has a good fit because the observed data set appears under the posterior predictive distribution (Supplementary Figure 3).

## Discussion

Contrary to our expectation, all clustering analyses concordantly showed no population structuring in the giant anteater across the large area studied. It is suggested that gene flow restriction among populations does not occur even considering that our sampling sites encompass different landscapes with high level of anthropic modifications. Therefore, this result must be taken with caution, since the studied area is under human-induced modification pressure, which can promote changes in the gene flow in long term.

It is well known the giant anteater demonstrates different ranges of movement throughout the Pantanal landscape from 1 km/day (Medri and Mourão, 2005) to 8 km/day in the Cerrado Biome (Bertassoni, 2010). It is likely that the absence of population structuring observed can be explained by this life trait and the biology of the species. The giant anteater has been observed living from highly conserved areas to anthropogenic areas, such as agricultural fields and wood plantations of *Pinus* sp., *Acacia* sp., and *Eucalyptus* sp. (Miranda, 2004; Braga, 2010; Vynne et al., 2011) and is therefore considered a species associated with several environments. In general, species associated with non-forested habitats may more easily cross the matrix and move between fragments, thereby reducing the negative effects of fragmentation-like genetic differentiation (Schlaepfer et al., 2018). The absence of spatial correlation between individuals, even when both sexes were separately analyzed, suggests that both sexes are similarly moving across the landscape. However, this capacity for moving across different landscape elements can make the individuals vulnerable to important threats for the species, such as human conflict and roadkill (Ng et al., 2008), which can explain why 60% of our sampled individuals were road-killed animals, promoting a significant loss of individuals in long term.

Moderate levels of genetic diversity (Ho = 0.54; He = 0.61) were observed in this large and single giant anteater population inhabiting the studied area. Similar values were previously reported for other local populations studied (Ho = 0.68, He = 0.72, Sartori et al., 2020; Ho = 0.60, He = 0.63, Garcia et al., 2005), suggesting that these can represent the mean values of genetic diversity along the distribution of the giant anteater. It is well known that genetic diversity has important ecological consequences in natural populations, including the maintenance of evolutionary potential and the individual ability to survive in response to threats as environmental changes and disease (Hughes et al., 2008). The combination of increased genetic drift, inbreeding, and restricted gene flow may substantially reduce the genetic variation of populations (Schlaepfer et al., 2018; Lino et al., 2019).

Lower genetic diversity has already been described in a small anteater population inhabiting a protected area (Ho = 0.059, He = 0.482), and it was associated with intense population reduction after recurrent fire events, resulting in inbreeding within the remaining individuals (Collevatti et al., 2007). An increased degree of homozygosity may cause the expression of deleterious recessive alleles, which can decrease individual fitness (Reed and Frankham, 2003). Our results found a significant inbreeding coefficient value (F_is_ = 0.13; *p* < 0.001) within the studied population, and it seems not biased either by a kinship effect. Since the *r* values found were very low, it is an indicative of a low level of relatedness among the individuals.

Besides the potential inbreeding detected, we also found a smaller mean number of effective alleles (Ae = 3.10) compared to the mean allele richness (Na = 5.5), suggesting that fewer alleles are contributing to maintain the current genetic diversity. These results can be a consequence of a Ne that is not large enough to retain all alleles in high frequency, since large Ne is necessary to retain more genetic diversity (Kimura and Crow, 1964).

The effective population size is an important factor that contributes to genetic variability maintenance because both heterozygosity and number of alleles are less impacted in populations with large effective size (Kimura and Crow, 1964; Reed and Frankham, 2003). It is known that effective population size varies with the generation time (Frankham, 1997; Reed and Frankham, 2003). A long generation time and lifespan can act as an intrinsic buffer against loss of genetic diversity (Hailer et al., 2006), resulting in a delayed detection of genetic diversity loss. The giant anteater lives from 20 to 30 years in captivity and has a long generation time (Nowak, 1991) and generation time (Desbiez et al., 2020); both biological features can explain a putative slow reduction in the genetic diversity found here. In species showing 1-year generation time, it is believed that Ne = 50 is enough to avoid the negative effects of inbreeding in the short term and Ne = 500 to prevent loss of variability by genetic drift in long term (Franklin, 1980; Soulé and Wilcox, 1980).

An effective population size Ne ≥ 1,000 was indicated for retaining the evolutionary potential for fitness in perpetuity (Frankham, 2015). Our results found Ne = 375 individuals in the studied area, a relatively high effective population size potentially extant in the studied region, highlighting the importance of this population for the conservation of giant anteaters. However, our demographic analyses suggested that the current giant anteater population has already suffered a recent bottleneck. Furthermore, the demographic history of the giant anteater population, inferred by a scenario test model and for the first time addressed here, also found a past reduction of the population size. Our inference from ABC analysis predicted past population size reduction.

Overall, our results showed a single and large population of giant anteaters inhabiting the southern edge of its geographical distribution, therefore already presenting negative genetic signals, as bottleneck and inbreeding, potentially caused by impacts of the increased human activities in the region. Of note, this work represents the study with the largest microsatellite set used in a Myrmecophagidae species, with a high polymorphic information content (PIC > 0.5), and the largest population genetic study thus far carried out in giant anteater, considering both the sampling area and number of individuals analyzed, reinforcing the importance of these results.

## Conservation Implications

For hundreds of years, the continuous impact of humans has been noticed in a decrease in the abundance and richness of organisms (Galetti and Dirzo, 2013). Our results suggest that the genetic consequences of these actions threaten the long-term population viability of *M. tridactyla* in the next 100 years. Despite the wide distribution of the species and the constant reports of threats for this animal, populations of giant anteater have been poorly studied in Brazil. It is important to highlight that conservation strategies should be urgently adopted to guarantee the species persistence. These strategies should be focused on reducing giant anteater mortality, by reducing the impacts such as road kills, hunting, and habitat loss (Bertassoni, 2012; Ascensão et al., 2016, 2019). Effective strategies would avoid population size reduction and ensure the maintenance of genetic diversity and the long-term viability of its populations that have been suffering mainly for the habitat loss.

## Data Availability Statement

The raw data supporting the conclusions of this article will be made available by the authors, without undue reservation.

## Ethics Statement

The animal study was reviewed and approved by the Ethics Committee on the Animal Experimentation (CEUA/UFSCar) protocol number 1584280817.

## Author Contributions

CB-R, PG, and AD designed research and data acquisition. CB-R, RS-S, BS, and PG contributed to new reagents or analytical tools. CB-R, RS-S, BS, AD, and PG analyzed the data, wrote, and review the manuscript. All authors contributed to the article and approved the submitted version.

## Conflict of Interest

The authors declare that the research was conducted in the absence of any commercial or financial relationships that could be construed as a potential conflict of interest.

## Footnotes

1 www.giantanteater.org
