# Supplementary material for: Moderate Genetic Diversity and Demographic Reduction in the Threatened Giant Anteater, Myrmecophaga tridactyla
Source: Front Genet. 2021 Jul 1;12:669350. doi: 10.3389/fgene.2021.669350 (PMC8280777; doi:10.3389/fgene.2021.669350)
Supplement: Supplementary file 1 [file Data_Sheet_1.docx]

Supplementary Material

Moderate genetic diversity and demographic reduction in the threatened giant anteater, *Myrmecophaga tridactyla*

## Supplementary Figures


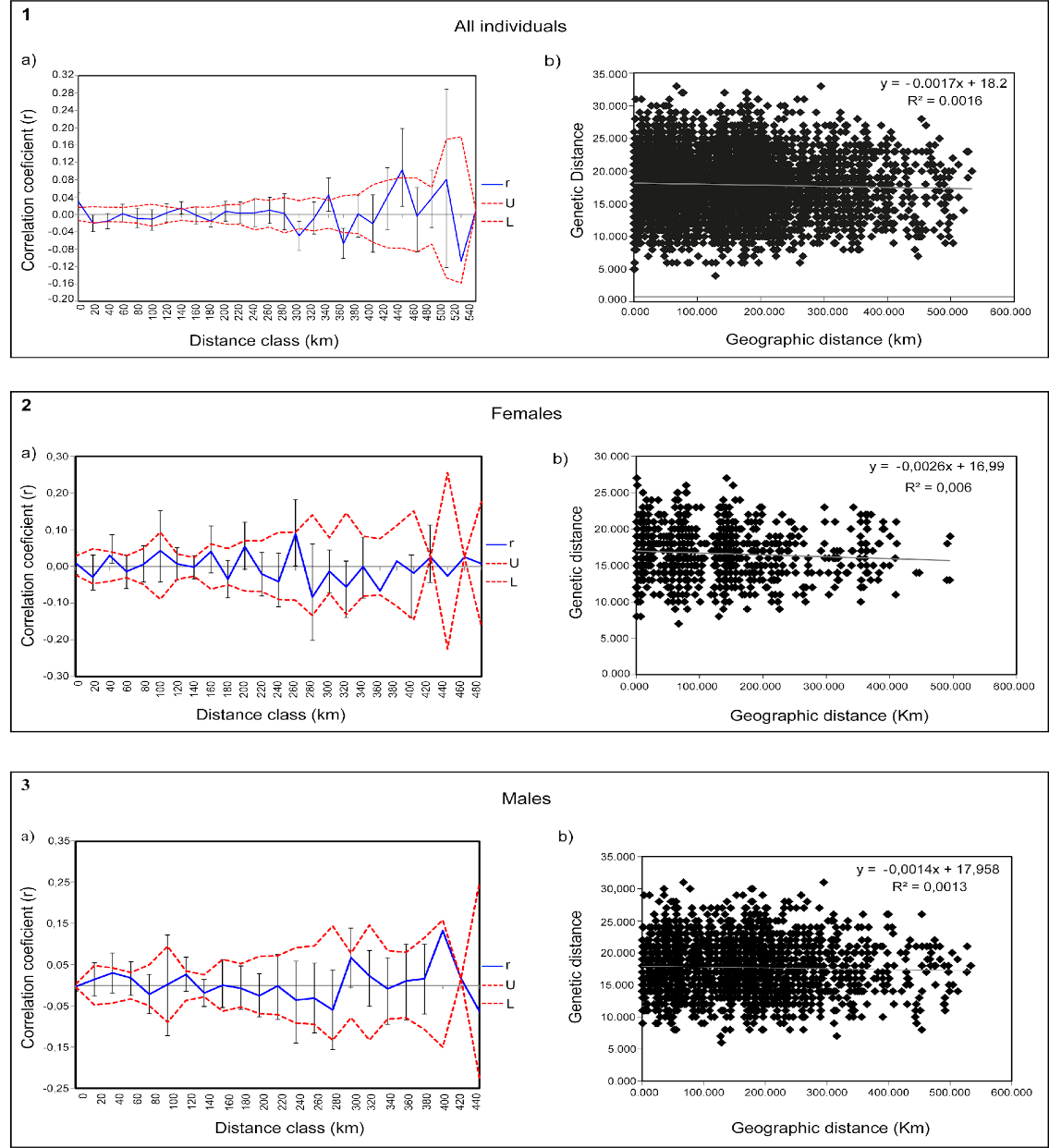


**Supplementary Figure 1.**Mantel test and spatial autocorrelation analysis results. In 1 (all individuals combined), 2 (40 females), and 3 (55 males). The correlograms (1a, 2a, and 3a) indicate the hierarchical spatial autocorrelation analysis showing the correlation coefficient r (solid blue line) as a function of geographic distance across defined spatial distance classes. Dashed red lines represent upper (U) and lower (L) bounds of the null hypothesis of no spatial structure based on 10000 random permutations. Error bars represent 95 % confidence intervals about r based on 1000 bootstraps. In 1b, 2b, and 3b, plotting pairwise codominant genotypic distance versus pairwise Euclidean distances (km) across all individuals, females, and males, respectively. Each point represents a pairwise comparison among individual giant anteater.


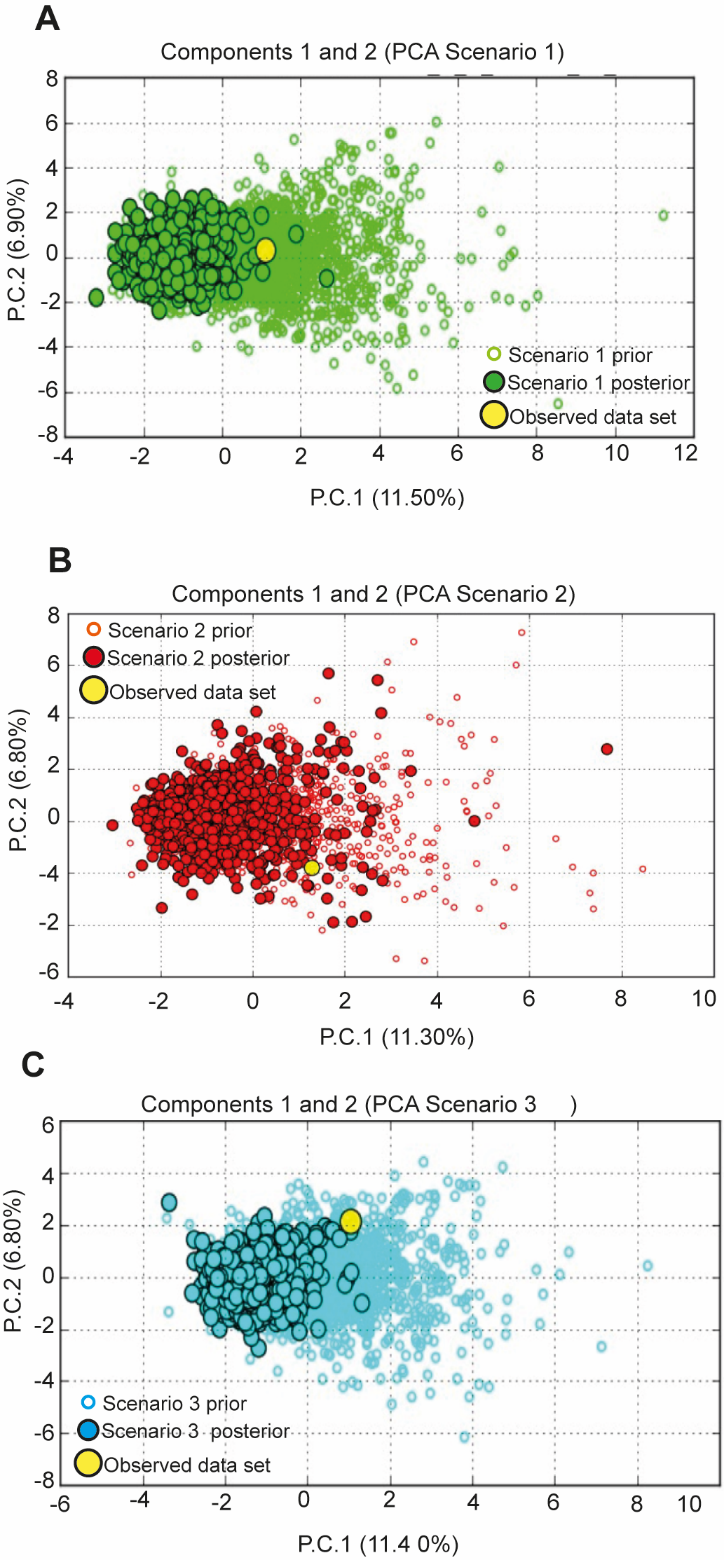


**Supplementary Figure 2.** Model checking by applying a PCA on the best-supported scenario (scenario 2) in DIYABC analysis. A. Graphic of Principal components analysis (PCA) generated in DYABC displaying the process checking scenario 1 demographic scenario for Myrmecophaga tridactyla. B. Graphic of PCA to check the best scenario 2. C. Graphic of PCA to check the scenario 3.


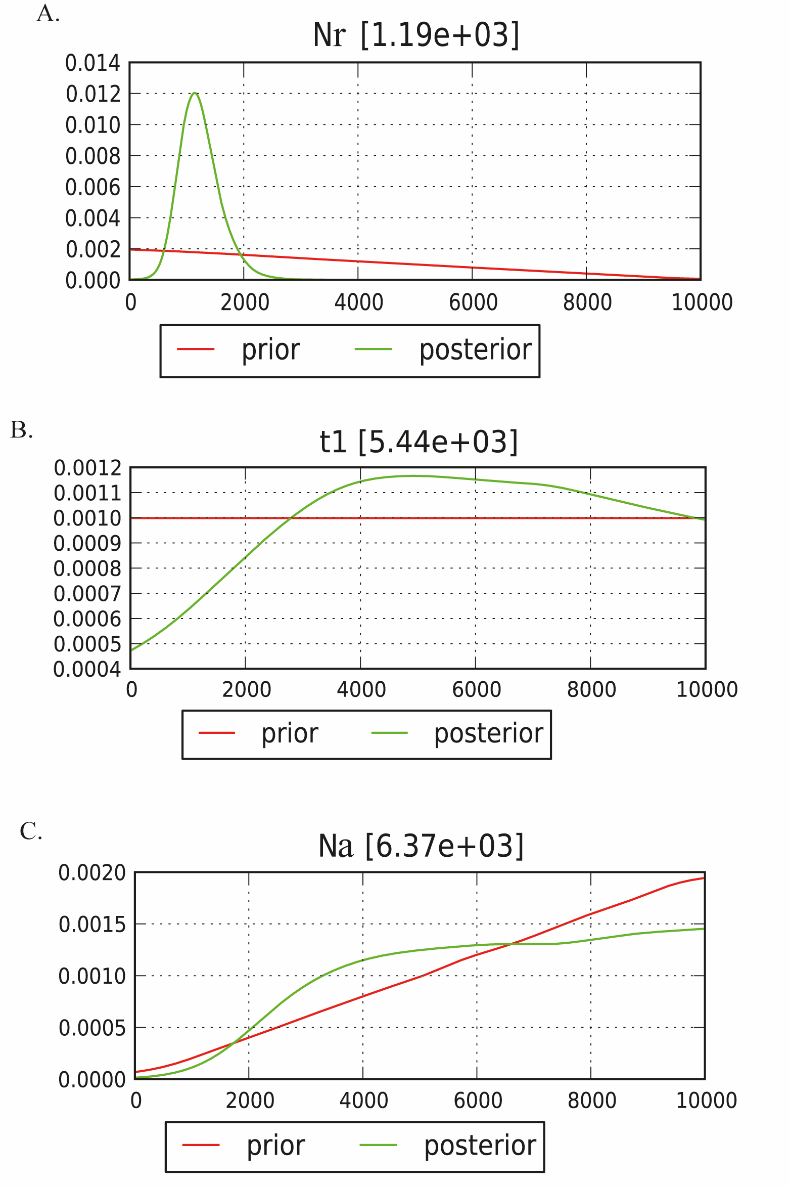


**Supplementary Figure 3.**Posterior distribution of the estimated parameters from the best model. A. Na, ancestral effective population size. B. Coalescent time t1 and C. Nr, is the recent effective population size.

## Supplementary Tables

**Supplementary Table 1.** Information about 107 Myrmecophaga tridactyla specimens analyzed in this study. For each individual, (ID - sample codes) we provided the following information: Sex (M-male, F- female), Type, CD (collection date), and localities (geographic coordinates).

| **ID** | **Sex** | **Type** | **CD** | **Latitude** | **Longitude** |
| --- | --- | --- | --- | --- | --- |
| 360 | M | Roadkill | April, 2013 | -20.8099701410031 | -54.5183188857456 |
| 554 | M | Roadkill | April, 2017 | -20.6836404823444 | -54.4231651812123 |
| 574 | M | Roadkill | April, 2017 | -21.6177577529032 | -53.6918530629973 |
| 581 | M | Roadkill | April, 2017 | -20.2490981059027 | -56.3553225935550 |
| 583 | M | Roadkill | April, 2017 | -21.9400850000000 | -53.3132610000000 |
| 404 | M | Roadkill | Dec, 2013 | -20.1673224597728 | -56.5573091854478 |
| 407 | M | Roadkill | Dec, 2013 | -20.3506323304339 | -56.1424640582395 |
| 414 | M | Roadkill | Feb, 2014 | -21.9497602292131 | -53.3161079518503 |
| 415 | M | Roadkill | Feb, 2014 | -20.7951707843327 | -51.8946594167791 |
| 417 | M | Roadkill | Feb, 2014 | -20.3577399265777 | -56.1271914299720 |
| 478 | M | Roadkill | Feb, 2017 | -21.6148799027978 | -53.7161476611256 |
| 490 | M | Roadkill | Feb, 2017 | -20.4726345526258 | -54.4095853414892 |
| 409 | M | Roadkill | Jan, 2014 | -21.6005080507981 | -53.8509697659370 |
| 476 | M | Roadkill | Jan, 2017 | -20.6836404823444 | -54.4231651812123 |
| 653 | M | Roadkill | July, 2017 | -20.4978644467484 | -54.2240606560014 |
| 622 | M | Roadkill | June, 2017 | -21.1379850000000 | -53.4910890000000 |
| 638 | M | Roadkill | June, 2017 | -20.6778548545702 | -52.2359984793259 |
| 363 | M | Roadkill | May, 2013 | -19.9122775452033 | -56.8609825659532 |
| 585 | M | Roadkill | May, 2017 | -20.7595639375226 | -52.0301198747727 |
| 587 | M | Roadkill | May, 2017 | -20.8736330000000 | -54.1415840000000 |
| 588 | M | Roadkill | May, 2017 | -21.1371003772023 | -53.4843089013385 |
| 510 | M | Roadkill | Mar, 2017 | -21.6017569245764 | -53.8360039244728 |
| 859 | M | Roadkill | Mar, 2018 | -20.5730450000000 | -52.4781540000000 |
| 401 | M | Roadkill | Nov, 2013 | -20.4887906125484 | -54.1362237957641 |
| 760 | M | Roadkill | Nov, 2017 | -20.4766930000000 | -54.3704190000000 |
| 387 | M | Roadkill | Oct, 2013 | -21.6276007575350 | -53.6170907192403 |
| 390 | M | Roadkill | Oct, 2013 | -20.1662898908932 | -56.5599437446914 |
| 393 | M | Roadkill | Oct, 2013 | -20.7329769183708 | -52.1166038355525 |
| 394 | M | Roadkill | Oct, 2013 | -20.7590069985773 | -52.0325943103135 |
| 704 | M | Roadkill | Oct, 2017 | -21.7815100000000 | -53.1876000000000 |
| 384 | M | Roadkill | Sep, 2013 | -20.1404902567460 | -56.6260453767553 |
| 386 | M | Roadkill | Sep, 2013 | -20.6797022696362 | -52.2315974568632 |
| 418 | M | Roadkill | Sep, 2013 | -21.6017569245764 | -53.8360039244728 |
| 757 | M | Roadkill | Sep, 2017 | -20.9074288693110 | -54.1335056613154 |
| 366 | F | Roadkill | May, 2013 | -20.7794692938385 | -51.9547930832546 |
| 375 | F | Roadkill | July, 2013 | -21.7476849755710 | -53.2776027716776 |
| 398 | F | Roadkill | Nov, 2013 | -21.6422679481749 | -53.4911107346586 |
| 485 | F | Roadkill | Feb, 2017 | -21.5881271911959 | -53.9653735773085 |
| 503 | F | Roadkill | Mar, 2017 | -20.1468345900431 | -56.6097447035794 |
| 509 | F | Roadkill | Mar, 2017 | -21.7133556457266 | -53.3396968290985 |
| 512 | F | Roadkill | Mar, 2017 | -20.4840520246359 | -52.7928715391113 |
| 556 | F | Roadkill | April, 2017 | -21.6148799027978 | -53.7161476611256 |
| 560 | F | Roadkill | April, 2017 | -20.3397304970204 | -56.1733133081723 |
| 572 | F | Roadkill | April, 2017 | -20.6915878177540 | -54.3742695738243 |
| 642 | F | Roadkill | June, 2017 | -21.1396145170700 | -53.5206722356191 |
| 767 | F | Roadkill | Nov, 2017 | -21.8256340000000 | -53.1078500000000 |
| 778 | F | Roadkill | Nov, 2017 | -21.1523698703842 | -53.0850531255658 |
| 851 | F | Roadkill | Feb, 2018 | -20.7908450000000 | -51.9107120000000 |
| 858 | F | Roadkill | Mar, 2018 | -20.5027500000000 | -52.6922870000000 |
| 364 | NA | Roadkill | May, 2013 | -20.8161302303806 | -54.5172096264141 |
| 471 | NA | Roadkill | Jan, 2017 | -20.4840520246359 | -52.7928715391113 |
| 553 | NA | Roadkill | Mar, 2017 | -20.4657879506312 | -54.0589371950263 |
| 595 | NA | Roadkill | May, 2017 | -20.4234444444000 | -54.9844722222000 |
| 472 | M | Roadkill | Jan, 2017 | -20.7969113980321 | -51.8883090795178 |
| 504 | M | Roadkill | Mar, 2017 | -20.4301739928254 | -54.8974364019231 |
| 552 | M | Roadkill | Mar, 2017 | -20.5683058875048 | -54.6339596969699 |
| 648 | M | Roadkill | July, 2017 | -20.4115856051861 | -54.7214056951203 |
| 659 | M | Roadkill | Aug, 2017 | -20.4686937000000 | -54.6741233000000 |
| 699 | M | Roadkill | Oct, 2017 | -20.8266095000000 | -54.1552321000000 |
| 843 | M | Roadkill | Feb, 2018 | -20.6839750000000 | -54.4252210000000 |
| 846 | M | Roadkill | Feb, 2018 | -20.5112800000000 | -54.5382180000000 |
| 507 | F | Roadkill | Mar, 2017 | -21.1746596917394 | -53.0225098801902 |
| 640 | F | Roadkill | June, 2017 | -20.6916498850332 | -54.2771147181092 |
| 646 | F | Roadkill | July, 2017 | -20.1440669643715 | -56.6168417030044 |
| 690 | F | Roadkill | Sep, 2017 | -20.1781830000000 | -54.9319920000000 |
| 664 | NA | Roadkill | Aug, 2017 | -21.6751157809633 | -53.4103505987422 |
| 539 | M | Capture | May, 2017 | -21.1590166667000 | -53.7572000000000 |
| 540 | M | Capture | May, 2017 | -21.1397833333000 | -53.7491666667000 |
| 543 | M | Capture | May, 2017 | -20.8106868867092 | -51.8349440158646 |
| 546 | M | Capture | June, 2017 | -20.4266418590310 | -55.0502387084270 |
| 549 | M | Capture | June, 2017 | -21.1377440951418 | -53.4890450590052 |
| 747 | M | Capture | July, 2017 | -21.1401355442286 | -53.7526143723369 |
| 903 | M | Capture | July, 2018 | -21.1132703349788 | -53.7497514584710 |
| 904 | M | Capture | May, 2018 | -21.6365541896063 | -53.5084142349243 |
| 906 | M | Capture | May, 2018 | -21.6410170519682 | -53.5923028620391 |
| 908 | M | Capture | May, 2018 | -21.6323548120860 | -53.4820372770272 |
| 911 | M | Capture | May, 2018 | -21.6303646641536 | -53.5039523060799 |
| 912 | M | Capture | May, 2018 | -21.6291261754413 | -53.5052538642057 |
| 916 | M | Capture | June, 2018 | -20.4830423408509 | -54.1511800383002 |
| 917 | M | Capture | June, 2018 | -20.4815936343173 | -54.0948089718569 |
| 918 | M | Capture | June,2018 | -20.4049617945771 | -53.8866298366285 |
| 919 | M | Capture | July, 2018 | -21.6321500000000 | -53.5021000000000 |
| 928 | M | Capture | Aug, 2018 | -20.4056100000000 | -53.9309000000000 |
| 929 | M | Capture | Aug, 2018 | -20.4258352000000 | -53.9661497000000 |
| 537 | F | Capture | May, 2017 | -21.0524000000000 | -53.9241333333000 |
| 538 | F | Capture | May, 2017 | -21.1396333333000 | -53.7513000000000 |
| 541 | F | Capture | May, 2017 | -20.4978644467484 | -54.2240606560014 |
| 547 | F | Capture | June, 2017 | -20.4613393195543 | -54.0361968586558 |
| 551 | F | Capture | June, 2017 | -21.6751157809633 | -53.4103505987422 |
| 745 | F | Capture | July, 2017 | -21.0463685492498 | -53.9214667134571 |
| 905 | F | Capture | May, 2018 | -21.6409638462326 | -53.5951312879058 |
| 907 | F | Capture | May, 2018 | -21.6608511915339 | -53.4374575509752 |
| 909 | F | Capture | May, 2018 | -21.6405560000000 | -53.4805560000000 |
| 915 | F | Capture | June, 2018 | -20.4912279034537 | -54.1024929654041 |
| 920 | F | Capture | July, 2018 | -21.6283600000000 | -53.5040800000000 |
| 921 | F | Capture | July, 2018 | -21.6342900000000 | -53.5185700000000 |
| 922 | F | Capture | July, 2018 | -21.6316100000000 | -53.5964300000000 |
| 930 | F | Capture | Aug, 2018 | -20.4609200000000 | -54.0326500000000 |
| 931 | F | Capture | Aug, 2018 | -20.4264700000000 | -53.9695900000000 |
| 932 | F | Capture | Aug, 2018 | -20.4742231000000 | -54.1059025000000 |
| 933 | F | Capture | Jan, 2019 | -21.6423170000000 | -53.5968370000000 |
| 934 | F | Capture | April, 2019 | -21.6444700000000 | -53.5989100000000 |
| 935 | F | Capture | April, 2019 | -21.6504700000000 | -53.6025700000000 |
| TB28 | F | Capture | May,2018 | -21.6255900000000 | -53.4877400000000 |
| TB29 | F | Capture | Oct,2019 | -21.6315640000000 | -53.4957860000000 |
| F2 | NA | Capture | 2019 | -21.6315640000000 | -53.4957860000000 |
| F3 | NA | Capture | 2019 | -21.6315640000000 | -53.4957860000000 |

| Locus name | Primer sequences 5'-3' | Repeat motif | T_a_ | Size range (bp) | Reference |
| --- | --- | --- | --- | --- | --- |
| 04 | R-TGTCTTCTTTACTCAGTGCTCC | (GT)_9_ | 62 | 172-180 | Garcia et al., 2005 |
|  | F-TGTAAAACGACGGCCAGTGGGTCAGATATCCTAATGGG |  |  |  |  |
| 07 | R-TGTGTCCTGTGAAGTAATGG | (GT)_42_ | 60 | 285-301 | Garcia et al., 2005 |
|  | F-TGTAAAACGACGGCCAGTAGGAGGATAAGATTAGGCAG |  |  |  |  |
| 11 | R-TCACCTTCATTGGAGCTTC | (GT)_15_ | 62 | 171-205 | Garcia et al., 2005 |
|  | F-TGTAAAACGACGGCCAGTTGTCTCTGTGTTAGGGTTCTTC |  |  |  |  |
| 13 | R-TGGTAAAGAATGAGGTC | (GT)_14_ | 58 | 238-248 | Garcia et al., 2005 |
|  | F-TGTAAAACGACGGCCAGTCTGCTCAGGTAACATTCC |  |  |  |  |
| 20 | R-CTATATGCTTGCCTTTGG | (GT)_14_ | 60 | 168-184 | Garcia et al., 2005 |
|  | F-TGTAAAACGACGGCCAGTCTTTCCTCATATCTCCCTG |  |  |  |  |
| A9 | R-TGTGAGCCACTGATCGTGTT | (AC)_8_ | 60 | 157-194 | Clozato et al., 2014 |
|  | F-TGTAAAACGACGGCCAGTTCCAAGTCTCAGGTCCCAT |  |  |  |  |
| B2 | R-AATGGTGGGGCACTAAGATG | (TG)_9_ | 60 | 191-233 | Clozato et al., 2014 |
|  | F-TGTAAAACGACGGCCAGTCCTTTGGGTCCTGATTGAGA |  |  |  |  |
| E3 | R-TGCTTACGCGTGGACAAAT | (GT)_23_ | 60 | 104-134 | Clozato et al., 2014 |
|  | F-TGTAAAACGACGGCCAGTCACCACGACACCACACTACC |  |  |  |  |
| G3 | R-TGGACTAACTGGGCTTCTGC | (GT)_18_ | 60 | 174-222 | Clozato et al., 2014 |
|  | F-TGTAAAACGACGGCCAGTTGGACCCGCCATATAAACAT |  |  |  |  |
| H5 | R-CCACGTCACAATCACCT | (TC)_6_ | 59 | 214-216 | Clozato et al., 2014 |
|  | F-TGTAAAACGACGGCCAGTCCCGCAGTATAGAAGCAG |  |  |  |  |

**Supplementary Table 2.** Microsatellite loci used in this study for *Myrmecophaga tridactyla*. Locus name, primer sequence R (reverse) and F (forward), the repeat motif, annealing temperature (T_a_), size of alleles and references. All forward sequences were labeled with M13.

**Supplementary Table 3.**Descriptions of prior setting for all parameters used in DIYABC. Ancestral effective population size (Na), recent population effective size (Nr), and coalescent time (t; in generations: 4 years for *M. tridactyla*; Nowak, 1991).

| Parameter | Min | Max | Parameter distribution |
| --- | --- | --- | --- |
| ***Effective population size*** | | |  |
| Na | 10 | 10000 | Uniform |
| Nr | 10 | 10000 | Uniform |
| ***Coalescent time scale in generations*** | | |  |
| t | 10 | 10000 | Uniform |
| ***Mutation model*** | |  |  |
| Mean mutation rate | 1x10^-4^ | 1x10^-3^ | - |

**Supplementary Table 4.** Relatedness estimators (*r*). QGM (Queller and Goodnight, 1989); RI (Ritland, 1996) andLRM (Lynch and Ritland, 1999) within each sampling sites.

|  | | **QGM** | **RI** | **LRM** |
| --- | --- | --- | --- | --- |
| Site 1 | ***Mean*** | -0.127 | -0.066 | -0.065 |
|  | ***SD*** | 0.336 | 0.080 | 0.082 |
| Site 2 | ***Mean*** | -0.036 | -0.020 | -0.018 |
|  | ***SD*** | 0.290 | 0.096 | 0.103 |
| Site 3 | ***Mean*** | -0.019 | -0.013 | -0.011 |
|  | ***SD*** | 0.322 | 0.094 | 0.114 |
| Site 4 | ***Mean*** | -0.096 | -0.046 | -0.046 |
|  | ***SD*** | 0.282 | 0.078 | 0.084 |

**Supplementary Table 5.**Summary of posterior probabilities of three demographic history scenarios evaluated in DIYABC analysis using microsatellite data.

| Scenarios | 1 | 2 | 3 |
| --- | --- | --- | --- |
| Posterior probabilities | 0.1568 | 0.8339 | 0.0092 |
| Confidence intervals | 0.1293-0.1844 | 0.8059-0.8620 | 0.0038-0.0146 |

**Supplementary Table 6.** Genotype information obtained from ten microsatellites for the 107 *Myrmecophaga tridactyla* specimens analyzed in this study. ID - sample codes; Pop –Sampling Sites. Zero indicating missing date.

| ID | Pop | 4 | 4 | 7 | 7 | 13 | 13 | 11 | 11 | 20 | 20 | A9 | A9 | B2 | B2 | E3 | E3 | G3 | G3 | H5 | H5 |
| --- | --- | --- | --- | --- | --- | --- | --- | --- | --- | --- | --- | --- | --- | --- | --- | --- | --- | --- | --- | --- | --- |
| 384 | 1 | 172 | 172 | 289 | 291 | 0 | 0 | 197 | 201 | 170 | 182 | 182 | 188 | 212 | 212 | 121 | 121 | 189 | 189 | 231 | 231 |
| 390 | 1 | 172 | 172 | 289 | 295 | 236 | 246 | 197 | 201 | 0 | 0 | 182 | 184 | 212 | 214 | 121 | 121 | 189 | 189 | 233 | 233 |
| 404 | 1 | 172 | 174 | 0 | 0 | 242 | 246 | 197 | 197 | 168 | 170 | 184 | 184 | 212 | 212 | 121 | 121 | 0 | 0 | 0 | 0 |
| 407 | 1 | 172 | 174 | 289 | 299 | 240 | 242 | 197 | 205 | 168 | 174 | 184 | 184 | 212 | 212 | 121 | 129 | 189 | 189 | 231 | 235 |
| 417 | 1 | 174 | 174 | 295 | 295 | 238 | 242 | 197 | 197 | 168 | 178 | 184 | 184 | 212 | 214 | 121 | 129 | 157 | 189 | 231 | 231 |
| 503 | 1 | 172 | 172 | 297 | 299 | 240 | 246 | 201 | 201 | 168 | 176 | 184 | 184 | 212 | 212 | 121 | 121 | 157 | 189 | 231 | 231 |
| 560 | 1 | 172 | 174 | 291 | 293 | 242 | 246 | 201 | 203 | 168 | 184 | 184 | 184 | 212 | 214 | 121 | 121 | 189 | 189 | 231 | 231 |
| 581 | 1 | 172 | 172 | 293 | 297 | 0 | 0 | 197 | 197 | 0 | 0 | 188 | 188 | 212 | 212 | 119 | 121 | 0 | 0 | 229 | 229 |
| 646 | 1 | 174 | 174 | 291 | 295 | 240 | 242 | 197 | 197 | 184 | 184 | 184 | 188 | 212 | 212 | 121 | 129 | 189 | 189 | 231 | 231 |
| 363 | 1 | 174 | 174 | 0 | 0 | 0 | 0 | 197 | 201 | 174 | 184 | 182 | 188 | 212 | 214 | 121 | 121 | 0 | 0 | 0 | 0 |
| 360 | 2 | 172 | 172 | 291 | 295 | 242 | 246 | 197 | 203 | 170 | 170 | 184 | 184 | 212 | 214 | 121 | 121 | 0 | 0 | 0 | 0 |
| 401 | 2 | 172 | 172 | 289 | 299 | 240 | 242 | 197 | 203 | 168 | 178 | 180 | 182 | 212 | 212 | 121 | 121 | 0 | 0 | 231 | 231 |
| 476 | 2 | 172 | 172 | 291 | 299 | 242 | 246 | 195 | 195 | 174 | 178 | 0 | 0 | 212 | 214 | 121 | 129 | 157 | 189 | 231 | 231 |
| 490 | 2 | 172 | 172 | 289 | 295 | 246 | 248 | 197 | 197 | 178 | 184 | 182 | 186 | 214 | 214 | 121 | 129 | 157 | 157 | 235 | 235 |
| 504 | 2 | 172 | 172 | 291 | 295 | 238 | 242 | 197 | 201 | 178 | 184 | 182 | 184 | 212 | 212 | 121 | 129 | 157 | 189 | 235 | 235 |
| 541 | 2 | 172 | 174 | 295 | 299 | 240 | 242 | 197 | 205 | 170 | 182 | 182 | 182 | 212 | 212 | 121 | 129 | 189 | 189 | 231 | 231 |
| 547 | 2 | 172 | 180 | 293 | 299 | 242 | 246 | 197 | 197 | 182 | 184 | 184 | 184 | 212 | 212 | 121 | 121 | 0 | 0 | 231 | 231 |
| 552 | 2 | 172 | 172 | 0 | 0 | 240 | 242 | 197 | 197 | 178 | 184 | 188 | 188 | 212 | 212 | 121 | 121 | 189 | 189 | 235 | 235 |
| 553 | 2 | 174 | 174 | 0 | 0 | 0 | 0 | 197 | 197 | 170 | 178 | 182 | 184 | 212 | 212 | 0 | 0 | 189 | 189 | 231 | 231 |
| 554 | 2 | 172 | 172 | 0 | 0 | 240 | 242 | 197 | 203 | 0 | 0 | 182 | 184 | 212 | 212 | 121 | 121 | 189 | 189 | 231 | 231 |
| 572 | 2 | 172 | 172 | 0 | 0 | 240 | 242 | 0 | 0 | 168 | 184 | 182 | 184 | 212 | 216 | 119 | 121 | 0 | 0 | 231 | 231 |
| 587 | 2 | 174 | 174 | 293 | 297 | 242 | 242 | 197 | 201 | 168 | 174 | 186 | 186 | 214 | 214 | 121 | 129 | 189 | 189 | 235 | 235 |
| 595 | 2 | 174 | 174 | 293 | 301 | 242 | 242 | 197 | 203 | 170 | 178 | 182 | 184 | 212 | 214 | 119 | 119 | 189 | 189 | 231 | 231 |
| 648 | 2 | 174 | 180 | 293 | 299 | 0 | 0 | 203 | 203 | 168 | 174 | 182 | 186 | 212 | 214 | 119 | 121 | 0 | 0 | 0 | 0 |
| 653 | 2 | 172 | 172 | 0 | 0 | 242 | 246 | 197 | 201 | 178 | 178 | 184 | 188 | 214 | 214 | 121 | 121 | 189 | 189 | 233 | 233 |
| 659 | 2 | 174 | 174 | 291 | 291 | 236 | 238 | 0 | 0 | 170 | 170 | 184 | 188 | 212 | 214 | 0 | 0 | 0 | 0 | 231 | 231 |
| 690 | 2 | 172 | 174 | 299 | 299 | 242 | 246 | 201 | 203 | 168 | 178 | 184 | 184 | 212 | 212 | 119 | 121 | 189 | 189 | 0 | 0 |
| 699 | 2 | 172 | 172 | 0 | 0 | 240 | 246 | 203 | 203 | 168 | 178 | 182 | 182 | 214 | 214 | 121 | 121 | 189 | 189 | 0 | 0 |
| 757 | 2 | 172 | 174 | 0 | 0 | 238 | 246 | 195 | 197 | 174 | 178 | 182 | 184 | 212 | 214 | 119 | 121 | 189 | 189 | 231 | 231 |
| 760 | 2 | 172 | 172 | 289 | 291 | 242 | 246 | 197 | 197 | 170 | 174 | 182 | 186 | 212 | 212 | 121 | 129 | 157 | 189 | 0 | 0 |
| 843 | 2 | 172 | 172 | 0 | 0 | 242 | 246 | 175 | 179 | 168 | 168 | 0 | 0 | 212 | 214 | 121 | 121 | 155 | 155 | 231 | 231 |
| 846 | 2 | 172 | 172 | 0 | 0 | 240 | 246 | 0 | 0 | 170 | 176 | 0 | 0 | 212 | 214 | 121 | 121 | 155 | 155 | 231 | 231 |
| 364 | 2 | 172 | 172 | 0 | 0 | 0 | 0 | 197 | 201 | 170 | 174 | 0 | 0 | 212 | 214 | 121 | 129 | 157 | 189 | 0 | 0 |
| 640 | 2 | 172 | 174 | 0 | 0 | 0 | 0 | 0 | 0 | 178 | 184 | 182 | 188 | 212 | 214 | 115 | 121 | 157 | 189 | 0 | 0 |
| 546 | 2 | 174 | 174 | 295 | 299 | 240 | 246 | 197 | 205 | 178 | 184 | 0 | 0 | 0 | 0 | 121 | 121 | 189 | 189 | 231 | 231 |
| 915 | 2 | 174 | 174 | 295 | 295 | 240 | 246 | 197 | 203 | 178 | 178 | 184 | 184 | 212 | 216 | 119 | 121 | 189 | 189 | 0 | 0 |
| 916 | 2 | 174 | 174 | 0 | 0 | 242 | 242 | 201 | 201 | 168 | 174 | 182 | 184 | 212 | 216 | 121 | 121 | 157 | 189 | 0 | 0 |
| 917 | 2 | 172 | 174 | 295 | 297 | 242 | 246 | 197 | 203 | 170 | 174 | 182 | 184 | 212 | 216 | 121 | 121 | 189 | 189 | 0 | 0 |
| 928 | 2 | 172 | 172 | 289 | 297 | 240 | 246 | 197 | 203 | 168 | 168 | 184 | 186 | 0 | 0 | 121 | 121 | 189 | 189 | 235 | 235 |
| 929 | 2 | 174 | 174 | 291 | 293 | 242 | 246 | 205 | 205 | 168 | 168 | 0 | 0 | 212 | 216 | 121 | 121 | 157 | 189 | 233 | 233 |
| 930 | 2 | 172 | 174 | 293 | 293 | 242 | 246 | 197 | 205 | 178 | 178 | 184 | 184 | 212 | 216 | 121 | 121 | 157 | 189 | 233 | 233 |
| 931 | 2 | 172 | 174 | 291 | 299 | 236 | 240 | 197 | 197 | 0 | 0 | 184 | 188 | 212 | 216 | 121 | 121 | 157 | 189 | 235 | 235 |
| 932 | 2 | 172 | 172 | 289 | 299 | 242 | 246 | 197 | 201 | 168 | 182 | 182 | 184 | 212 | 216 | 121 | 121 | 0 | 0 | 233 | 235 |
| 918 | 2 | 172 | 174 | 0 | 0 | 240 | 246 | 0 | 0 | 168 | 168 | 0 | 0 | 0 | 0 | 121 | 121 | 157 | 189 | 231 | 231 |
| 375 | 3 | 172 | 172 | 287 | 295 | 242 | 242 | 197 | 201 | 0 | 0 | 0 | 0 | 212 | 216 | 121 | 129 | 0 | 0 | 231 | 231 |
| 387 | 3 | 172 | 174 | 291 | 295 | 238 | 240 | 197 | 197 | 176 | 178 | 182 | 188 | 212 | 212 | 119 | 119 | 189 | 189 | 231 | 231 |
| 409 | 3 | 174 | 178 | 297 | 297 | 240 | 242 | 197 | 201 | 170 | 174 | 188 | 188 | 212 | 214 | 121 | 121 | 0 | 0 | 231 | 231 |
| 414 | 3 | 174 | 178 | 0 | 0 | 240 | 242 | 197 | 197 | 168 | 176 | 184 | 184 | 212 | 214 | 121 | 121 | 189 | 189 | 231 | 235 |
| 418 | 3 | 172 | 174 | 299 | 299 | 240 | 240 | 197 | 197 | 178 | 178 | 182 | 184 | 0 | 0 | 121 | 121 | 157 | 189 | 231 | 231 |
| 478 | 3 | 172 | 172 | 0 | 0 | 242 | 246 | 197 | 201 | 170 | 176 | 182 | 182 | 212 | 212 | 121 | 129 | 0 | 0 | 231 | 231 |
| 485 | 3 | 172 | 172 | 289 | 295 | 242 | 242 | 197 | 201 | 178 | 178 | 184 | 188 | 212 | 214 | 121 | 121 | 157 | 189 | 0 | 0 |
| 507 | 3 | 172 | 172 | 0 | 0 | 0 | 0 | 197 | 203 | 174 | 184 | 182 | 184 | 212 | 212 | 121 | 121 | 157 | 157 | 231 | 231 |
| 509 | 3 | 174 | 174 | 297 | 299 | 242 | 248 | 201 | 205 | 170 | 174 | 184 | 184 | 212 | 216 | 121 | 121 | 157 | 157 | 231 | 231 |
| 510 | 3 | 172 | 174 | 295 | 297 | 238 | 242 | 197 | 205 | 170 | 174 | 182 | 182 | 212 | 214 | 121 | 121 | 157 | 189 | 231 | 231 |
| 537 | 3 | 172 | 172 | 289 | 291 | 242 | 242 | 0 | 0 | 168 | 174 | 182 | 184 | 212 | 214 | 121 | 129 | 0 | 0 | 0 | 0 |
| 538 | 3 | 174 | 174 | 289 | 293 | 236 | 238 | 197 | 201 | 170 | 178 | 184 | 184 | 212 | 214 | 121 | 121 | 157 | 189 | 0 | 0 |
| 539 | 3 | 172 | 174 | 0 | 0 | 240 | 242 | 197 | 205 | 174 | 174 | 182 | 182 | 212 | 212 | 121 | 129 | 189 | 189 | 231 | 231 |
| 540 | 3 | 172 | 172 | 0 | 0 | 240 | 248 | 197 | 205 | 170 | 180 | 188 | 188 | 212 | 212 | 121 | 121 | 0 | 0 | 231 | 231 |
| 549 | 3 | 174 | 178 | 291 | 297 | 246 | 246 | 201 | 205 | 174 | 184 | 188 | 188 | 212 | 214 | 121 | 121 | 189 | 189 | 231 | 231 |
| 551 | 3 | 172 | 172 | 0 | 0 | 240 | 242 | 197 | 197 | 174 | 178 | 182 | 188 | 212 | 214 | 121 | 121 | 189 | 189 | 231 | 231 |
| 556 | 3 | 172 | 172 | 285 | 299 | 0 | 0 | 0 | 0 | 178 | 184 | 182 | 184 | 212 | 214 | 121 | 121 | 0 | 0 | 231 | 231 |
| 574 | 3 | 172 | 172 | 0 | 0 | 242 | 246 | 203 | 203 | 0 | 0 | 184 | 186 | 212 | 212 | 121 | 121 | 157 | 189 | 231 | 231 |
| 583 | 3 | 174 | 174 | 291 | 299 | 242 | 246 | 201 | 201 | 168 | 178 | 184 | 188 | 212 | 214 | 121 | 129 | 157 | 189 | 235 | 235 |
| 588 | 3 | 174 | 174 | 291 | 301 | 242 | 242 | 197 | 205 | 178 | 178 | 184 | 186 | 212 | 212 | 119 | 121 | 189 | 189 | 235 | 235 |
| 622 | 3 | 172 | 172 | 293 | 297 | 242 | 248 | 197 | 197 | 174 | 178 | 184 | 188 | 212 | 212 | 121 | 121 | 189 | 189 | 231 | 231 |
| 642 | 3 | 172 | 172 | 289 | 291 | 238 | 246 | 197 | 205 | 176 | 184 | 186 | 186 | 212 | 214 | 119 | 121 | 157 | 189 | 231 | 231 |
| 664 | 3 | 172 | 172 | 0 | 0 | 238 | 242 | 197 | 205 | 170 | 176 | 188 | 188 | 212 | 212 | 121 | 121 | 189 | 189 | 233 | 233 |
| 704 | 3 | 172 | 172 | 289 | 299 | 242 | 242 | 201 | 203 | 174 | 184 | 182 | 184 | 212 | 214 | 121 | 121 | 0 | 0 | 231 | 231 |
| 745 | 3 | 174 | 174 | 291 | 291 | 240 | 242 | 203 | 205 | 174 | 178 | 186 | 186 | 214 | 216 | 121 | 129 | 157 | 189 | 231 | 231 |
| 747 | 3 | 172 | 174 | 289 | 295 | 236 | 238 | 197 | 197 | 178 | 184 | 182 | 184 | 212 | 214 | 121 | 121 | 157 | 189 | 231 | 231 |
| 767 | 3 | 174 | 180 | 289 | 293 | 238 | 246 | 197 | 197 | 168 | 178 | 182 | 184 | 212 | 212 | 121 | 129 | 189 | 189 | 231 | 231 |
| 778 | 3 | 172 | 172 | 289 | 289 | 242 | 246 | 201 | 203 | 174 | 184 | 0 | 0 | 212 | 212 | 121 | 129 | 157 | 189 | 231 | 231 |
| 398 | 3 | 172 | 172 | 0 | 0 | 242 | 246 | 0 | 0 | 170 | 174 | 188 | 188 | 212 | 212 | 121 | 129 | 0 | 0 | 0 | 0 |
| 905 | 3 | 174 | 174 | 289 | 291 | 238 | 246 | 0 | 0 | 0 | 0 | 0 | 0 | 212 | 212 | 121 | 121 | 189 | 189 | 231 | 231 |
| 906 | 3 | 172 | 174 | 289 | 299 | 242 | 246 | 201 | 203 | 178 | 178 | 0 | 0 | 212 | 214 | 119 | 121 | 189 | 189 | 231 | 231 |
| 907 | 3 | 172 | 172 | 297 | 299 | 242 | 246 | 197 | 201 | 168 | 174 | 0 | 0 | 214 | 214 | 121 | 121 | 157 | 189 | 0 | 0 |
| 909 | 3 | 174 | 174 | 291 | 297 | 240 | 242 | 197 | 201 | 178 | 184 | 0 | 0 | 0 | 0 | 119 | 121 | 157 | 189 | 0 | 0 |
| 912 | 3 | 172 | 174 | 291 | 297 | 236 | 238 | 197 | 205 | 170 | 170 | 0 | 0 | 0 | 0 | 119 | 119 | 157 | 189 | 0 | 0 |
| 919 | 3 | 172 | 172 | 0 | 0 | 240 | 246 | 197 | 203 | 168 | 170 | 184 | 184 | 212 | 216 | 0 | 0 | 189 | 189 | 0 | 0 |
| 920 | 3 | 174 | 174 | 289 | 291 | 236 | 242 | 197 | 197 | 178 | 178 | 0 | 0 | 212 | 216 | 121 | 121 | 157 | 189 | 231 | 231 |
| 921 | 3 | 172 | 178 | 289 | 293 | 240 | 240 | 197 | 203 | 170 | 178 | 186 | 186 | 212 | 216 | 121 | 121 | 157 | 189 | 225 | 225 |
| 922 | 3 | 174 | 174 | 291 | 295 | 238 | 242 | 205 | 205 | 178 | 178 | 182 | 184 | 212 | 216 | 121 | 121 | 157 | 189 | 225 | 231 |
| 933 | 3 | 174 | 174 | 295 | 299 | 238 | 242 | 0 | 0 | 178 | 184 | 184 | 188 | 212 | 216 | 0 | 0 | 189 | 189 | 233 | 233 |
| 934 | 3 | 172 | 172 | 295 | 297 | 242 | 246 | 171 | 179 | 170 | 178 | 184 | 184 | 212 | 216 | 119 | 121 | 189 | 189 | 233 | 233 |
| 935 | 3 | 172 | 172 | 297 | 299 | 238 | 246 | 197 | 205 | 168 | 168 | 182 | 184 | 212 | 216 | 121 | 121 | 189 | 189 | 0 | 0 |
| F2 | 3 | 172 | 172 | 295 | 299 | 240 | 242 | 197 | 197 | 170 | 174 | 0 | 0 | 0 | 0 | 121 | 121 | 157 | 189 | 233 | 233 |
| F3 | 3 | 172 | 172 | 295 | 299 | 240 | 242 | 197 | 197 | 170 | 184 | 0 | 0 | 212 | 216 | 121 | 129 | 157 | 189 | 233 | 233 |
| TB28 | 3 | 172 | 172 | 289 | 293 | 242 | 246 | 197 | 203 | 170 | 184 | 0 | 0 | 212 | 216 | 121 | 121 | 157 | 189 | 233 | 233 |
| TB29 | 3 | 172 | 172 | 0 | 0 | 242 | 246 | 0 | 0 | 170 | 184 | 0 | 0 | 212 | 216 | 121 | 121 | 157 | 189 | 233 | 233 |
| 903 | 3 | 174 | 174 | 291 | 295 | 242 | 242 | 201 | 205 | 0 | 0 | 0 | 0 | 212 | 214 | 0 | 0 | 189 | 189 | 0 | 0 |
| 904 | 3 | 172 | 172 | 0 | 0 | 242 | 242 | 197 | 197 | 174 | 184 | 0 | 0 | 212 | 212 | 121 | 121 | 0 | 0 | 0 | 0 |
| 908 | 3 | 172 | 172 | 295 | 297 | 238 | 242 | 201 | 201 | 0 | 0 | 0 | 0 | 0 | 0 | 119 | 121 | 157 | 189 | 0 | 0 |
| 911 | 3 | 172 | 172 | 0 | 0 | 242 | 242 | 197 | 201 | 170 | 184 | 0 | 0 | 0 | 0 | 121 | 121 | 157 | 189 | 0 | 0 |
| 366 | 4 | 172 | 172 | 289 | 291 | 240 | 246 | 197 | 205 | 176 | 178 | 184 | 188 | 212 | 214 | 121 | 121 | 0 | 0 | 0 | 0 |
| 393 | 4 | 172 | 178 | 0 | 0 | 242 | 242 | 197 | 203 | 174 | 184 | 188 | 188 | 212 | 212 | 129 | 129 | 189 | 189 | 231 | 231 |
| 394 | 4 | 172 | 174 | 0 | 0 | 242 | 242 | 197 | 203 | 174 | 184 | 184 | 184 | 212 | 214 | 0 | 0 | 189 | 189 | 231 | 231 |
| 415 | 4 | 172 | 174 | 293 | 293 | 242 | 246 | 197 | 197 | 168 | 178 | 182 | 184 | 212 | 214 | 121 | 121 | 157 | 189 | 231 | 231 |
| 471 | 4 | 172 | 172 | 0 | 0 | 240 | 242 | 197 | 197 | 0 | 0 | 182 | 184 | 212 | 216 | 121 | 121 | 157 | 189 | 235 | 235 |
| 472 | 4 | 172 | 172 | 291 | 299 | 242 | 246 | 197 | 201 | 168 | 178 | 184 | 184 | 212 | 214 | 121 | 129 | 157 | 189 | 233 | 233 |
| 512 | 4 | 172 | 172 | 0 | 0 | 240 | 242 | 197 | 201 | 170 | 174 | 182 | 184 | 212 | 214 | 0 | 0 | 157 | 189 | 231 | 231 |
| 543 | 4 | 174 | 174 | 289 | 293 | 242 | 242 | 197 | 205 | 170 | 182 | 186 | 188 | 212 | 214 | 121 | 129 | 189 | 189 | 231 | 231 |
| 585 | 4 | 174 | 174 | 291 | 291 | 236 | 242 | 195 | 197 | 168 | 174 | 182 | 182 | 212 | 212 | 121 | 129 | 0 | 0 | 233 | 233 |
| 638 | 4 | 174 | 174 | 289 | 299 | 242 | 246 | 197 | 197 | 170 | 174 | 184 | 188 | 212 | 212 | 121 | 121 | 0 | 0 | 231 | 231 |
| 851 | 4 | 172 | 172 | 291 | 299 | 238 | 238 | 197 | 205 | 174 | 184 | 184 | 184 | 212 | 214 | 121 | 121 | 157 | 157 | 231 | 231 |
| 858 | 4 | 174 | 174 | 295 | 299 | 238 | 246 | 197 | 205 | 170 | 178 | 0 | 0 | 212 | 214 | 121 | 121 | 0 | 0 | 0 | 0 |
| 859 | 4 | 174 | 180 | 299 | 299 | 242 | 246 | 197 | 201 | 168 | 184 | 0 | 0 | 212 | 214 | 119 | 121 | 157 | 157 | 231 | 231 |
| 386 | 4 | 172 | 172 | 0 | 0 | 238 | 242 | 201 | 201 | 168 | 174 | 180 | 184 | 212 | 212 | 0 | 0 | 0 | 0 | 0 | 0 |
